# Supplementary material for: A review and evaluation of secondary school accountability in England: Statistical strengths, weaknesses and challenges for ‘Progress 8’ raised by COVID‐19
Source: Rev Educ. 2021 Aug 27;9(3):e3299. doi: 10.1002/rev3.3299 (PMC8661617; doi:10.1002/rev3.3299)
Supplement: Supplementary file 2 — Supplementary Material [file REV3-9-0-s001.docx]

# Supplementary Material

In this section we provide technical information on the calculation of a school’s Progress 8 score (Department for Education, 2020a), including how the Progress 8 methodology can be formulated as an application of linear regression.

Let $y_{ij}$ and $x_{ij}$ denote the Attainment 8 and KS2 score of pupil $i$ ($i=1,\ldots,n_{j}$) in school $j$ ($j=1,\ldots,J$). A pupil’s Progress 8 score is calculated as the difference between their Attainment 8 score and the average Attainment 8 scores among all pupils nationally who had the same prior attainment as measured by pupils’ average KS2 test scores in reading and maths. This difference is divided by 10 as school Progress 8 is reported on a per subject basis (recall that while Progress 8 covers eight qualifications, English and Maths GCSE grades are double counted). Let $p_{ij}$ denote the resulting pupil Progress 8 score.

The school Progress 8 score $\bar{p}_{.j}$ is then calculated as the average pupil Progress 8 score in each school.

$\bar{p}_{.j}=\frac{1}{n_{j}}\sum_{i=1}^{n_{j}} p_{ij}$ (1)

From 2018 onwards, the DfE limited how negative a pupil’s progress 8 score could be to help stop distortion of the overall performance of a school. Minimum thresholds are set for each prior attainment group, and pupils whose scores are below these thresholds have their scores altered to be set at the minimum. The threshold is determined by a set number of standard deviations below the mean for each prior attainment group so that approximately 1% of pupils are identified for adjustment nationally.

The lower and upper limits of the 95% confidence intervals for the school Progress 8 scores are calculated as

$\hat{\mathrm{LL}}\left( \hat{p}_{j} \right)=\bar{p}_{.j}-1.96\times\hat{\mathrm{SE}}\left( \bar{p}_{.j} \right)$ (2)

$\hat{\mathrm{UL}}\left( \hat{p}_{j} \right)=\bar{p}_{.j}+1.96\times\hat{\mathrm{SE}}\left( \bar{p}_{.j} \right)$ (3)

where 1.96 is the critical value for the 95% confidence intervals and $\hat{\mathrm{SE}}\left( \bar{p}_{.j} \right)$ is the standard error associated with the school Progress 8 score. The latter is calculated as

$\hat{\mathrm{SE}}\left( \hat{p}_{j} \right)=\frac{\hat{\sigma}}{\sqrt{n_{j}}}$ (4)

where $\hat{\sigma}$ donates the standard deviation of pupils’ Progress 8 scores nationally

$\hat{\sigma}=\sqrt{\frac{\sum_{i=1}^{N} \left( p_{ij}-\bar{p}.. \right)^{2}}{N-1}}$ (5)

Progress 8 bandings are then calculated as follows

| Banding | Definition | |
| --- | --- | --- |
|  | Score | Significant |
| 5 = Well above average | $0.5\leq\bar{p}_{.j}$ | Yes if $\hat{\mathrm{LL}}\left( \hat{p}_{j} \right)>0$ |
| 4 = Above average | $0<\bar{p}_{.j}<0.5$ | Yes if $\hat{\mathrm{LL}}\left( \hat{p}_{j} \right)>0$ |
| 3 = Average | Any | No if $\hat{\mathrm{LL}}\left( \hat{p}_{j} \right)<0 \&\hat{\mathrm{UL}}\left( \hat{p}_{j} \right)>0$ |
| 2 = Below average | $0.5<\bar{p}_{.j}<0$ | Yes if $\hat{\mathrm{UL}}\left( \hat{p}_{j} \right)<0$ |
| 1 = Well below average | $\bar{p}_{.j}\leq-0.5$ | Yes if $\hat{\mathrm{UL}}\left( \hat{p}_{j} \right)<0$ |

We note in passing two oddities with the way the 95% confidence intervals are calculated. First, the use of 1.96 for the 95% confidence intervals implicitly assumes that school cohorts are very large whereas they are rather small (the average school cohort consists of approximately 160 pupils). Correcting for this would slightly widen the 95% confidence intervals, especially for small schools. For example, while 160 pupils implies a multiplier of 1.97, just 10 pupils implies a multiplier of 2.23. Second, the definition of $\hat{\sigma}$ is the standard deviation of pupil Progress 8 scores nationally whereas a more usual choice would be to define it as the standard deviation of pupil Progress 8 scores within the school under consideration, or perhaps the average across all within-school standard deviations. The latter is smaller than the standard deviation of pupil Progress 8 scores nationally. Correcting for this would slightly narrow the 95% confidence intervals. As the two corrections work in opposite directions, they will to some extent cancel each other out.

In Leckie and Goldstein (2019) we noted that we can formulate the Progress 8 methodology as an application of conventional linear regression. Namely a linear regression of $y_{ij}$ on $x_{ij}$ where we enter $x_{ij}$ as a series of 34 dummy variables $x_{1ij},\ldots,x_{34ij}$, one for each unique value of $x_{ij}$ and where we have omitted the usual constant term. This is an important insight as most school value-added performance measures are instead derived using multilevel linear regression and the two approaches lead to different estimates of school performance and in some scenarios these differences may be qualitatively important (see Section 6). A further benefit of formulating the Progress 8 methodology as an application of conventional linear regression is that we can then apply any of the usual extensions possible in linear regression, for example, it is easy to see how adjustments can be made for pupil demographic and socioeconomic background characteristics and their interactions simply by adding them as additional covariates (Section 4).

The linear regression can be written as

$y_{ij}=\beta_{1}x_{1ij}+\cdots+\beta_{34}x_{34ij}+r_{ij}$ (6)

The regression coefficients $\beta_{1},\ldots,\beta_{34}$ measure the expected value of $y_{ij}$ for each value of $x_{ij}$. In other words, the national average Attainment 8 score for pupils at each observed value of KS2 prior attainment.

Fitting this model by ordinary least-squares allows us to estimate these values $\hat{\beta}_{1},\ldots,\hat{\beta}_{34}$. We can then assign these values to pupils via the usual linear regression prediction equation

$\hat{y}_{ij}=\hat{\beta}_{1}x_{1ij}+\cdots+\hat{\beta}_{34}x_{34ij}$ (7)

where $\hat{y}_{ij}$ denotes the predicted Attainment 8 score for each pupil (the average Attainment 8 scores among all pupils nationally who had the same prior attainment).

The pupil Progress 8 score is then calculated as the difference between each pupil’s actual Attainment 8 score $y_{ij}$ and their predicted Attainment 8 scores $\hat{y}_{ij}$. This difference is as before divided by 10. The pupil Progress 8 score $p_{ij}$ is therefore equal to the predicted residual from the above linear regression $\hat{r}_{ij}$ divided by 10. The school Progress 8 scores are once again the school averages of the pupil Progress 8 scores.

From 2018 onwards, the DfE limited how negative a pupil’s progress 8 score could be to help stop distortion of the overall performance of a school. Minimum thresholds are set for each prior attainment group, and pupils whose scores are below these thresholds have their scores altered to be set at the minimum. The threshold is determined by a set number of standard deviations below the mean for each prior attainment group so that approximately 1% of pupils are identified for adjustment nationally.

Further information on Progress 8 is provided in the following documents:

https://assets.publishing.service.gov.uk/government/uploads/system/uploads/attachment_data/file/872997/Secondary_accountability_measures_guidance_February_2020_3.pdf

https://assets.publishing.service.gov.uk/government/uploads/system/uploads/attachment_data/file/561021/Progress_8_and_Attainment_8_how_measures_are_calculated.pdf
